# Liquiritigenin attenuates isoprenaline-induced myocardial fibrosis in mice through the TGF-β1/Smad2 and AKT/ERK signaling pathways

**DOI:** 10.3892/mmr.2021.12326

**Published:** 2021-07-30

**Authors:** Li Li, Hui Fang, Yong-Hong Yu, Shan-Xin Liu, Zhi-Qiang Yang

**Affiliations:** 1 Department of Ultrasonography, Tongde Hospital of Zhejiang Province, Hangzhou, Zhejiang 310012, P.R. China; 2 Department of Cardiology, The Affiliated Hospital of Hangzhou Normal University, Hangzhou, Zhejiang 310000, P.R. China; 3 Type-B Ultrasonic Room, Heart Center, The Affiliated Hospital of Hangzhou Normal University, Hangzhou, Zhejiang 310000, P.R. China

**Keywords:** extracellular signal-regulated kinase, liquiritigenin, myocardial fibrosis, protein kinase B, Smad2, transforming growth factor β1, ultrasound imaging

## Abstract

Myocardial fibrosis is a pathological process characterized by excessive accumulation of extracellular matrix in myocardial interstitial spaces. Myocardial fibrosis is a fundamental process in ventricular remodeling and a primary contributor to the progression of heart failure. Liquiritigenin (LQ) is a flavanone compound with anti-oxidative, anti-carcinogenic, anti-inflammatory and estrogenic properties. The present study aimed to investigate the regulatory potential of LQ treatment in a mouse model of isoprenaline (ISO)-induced cardiac fibrosis and in cultured H9C2 cardiomyocytes stimulated with angiotensin II (Ang II). The treatment of ISO-induced mice with LQ significantly decreased the levels of cardiac injury-related proteins in the serum and ECM accumulation in mouse heart tissues. LQ treatment also effectively alleviated cardiac dysfunction in ISO-treated mice. Further analyses revealed that LQ inhibited ISO-induced collagen formation and activation of the transforming growth factor-β1 (TGF-β1)/Smad2 and protein kinase B (AKT)/extracellular signal-regulated kinase (ERK) signaling pathways. As a major pathological event in myocardial fibrosis, the apoptosis of cardiomyocytes has been considered a key mechanism contributing to impaired left ventricle performance. The pretreatment of rat cardiomyocytes with LQ significantly reduced the apoptosis of H9C2 cells, and inhibited Ang II-induced activation of the TGF-β1/Smad2 and AKT/ERK pathways. In conclusion, the present study revealed that LQ ameliorated ISO-induced myocardial fibrosis in mice and inhibited the apoptosis of cardiomyocytes *in vitro* by inhibiting the TGF-β1/Smad2 and AKT/ERK signaling pathways. These results suggested the anti-fibrotic and cardioprotective potential of LQ in fibrosis, thus supporting the use of LQ for the management of cardiomyocyte injury and myocardial fibrosis in patients with cardiac diseases.

## Introduction

The myocardial extracellular matrix (ECM) is an intricate and dynamic network consisting of extracellular proteins that provides structural and functional support for the myocardium (1). The disruption of ECM homeostasis is a primary driver for the development of cardiac dysfunction and heart failure (2). Myocardial fibrosis is a pathological process characterized by excessive accumulation of ECM in myocardial interstitial spaces and has been recognized as a common feature in various cardiac diseases (3). The myocardial architecture is disorganized during myocardial fibrosis, which facilitates the progression of cardiac dysfunction, myocardial infarction, ventricular arrhythmias and eventually heart failure (4). Thus, an effective and safe therapy that can inhibit myocardial fibrosis from progressing is critical to prevent heart failure in patients with cardiac diseases (5).

Transforming growth factor-β1 (TGF-β1) is a central fibrogenic factor that has been reported to increase ECM expression, induce the transformation of fibroblasts to myofibroblasts, and mediate the production of pro-fibrotic cytokines during myocardial fibrosis (6). Upregulated TGF-β1 can bind to the type II receptor, which phosphorylates the type I receptor, leading to translocation of the Smad heterotrimeric complex into the nucleus and the activation of the Smad-dependent pathway (7). The protein kinase B (AKT)/extracellular signal-regulated kinase (ERK) signaling pathway is also downstream of TGF-β1 and can be activated during cardiac fibrosis (8). TGF-β1 has been reported to trigger phosphorylation of the AKT pathway in a RhoA-dependent manner, whereas it induces the activation of ERK via small GTPase or direct phosphorylation of the ShcA protein (9). As no efficient anti-fibrotic treatment is currently available, the exploration of novel therapeutic approaches targeting these molecular pathways may provide insights into the management of myocardial fibrosis.

**Figure 1.**
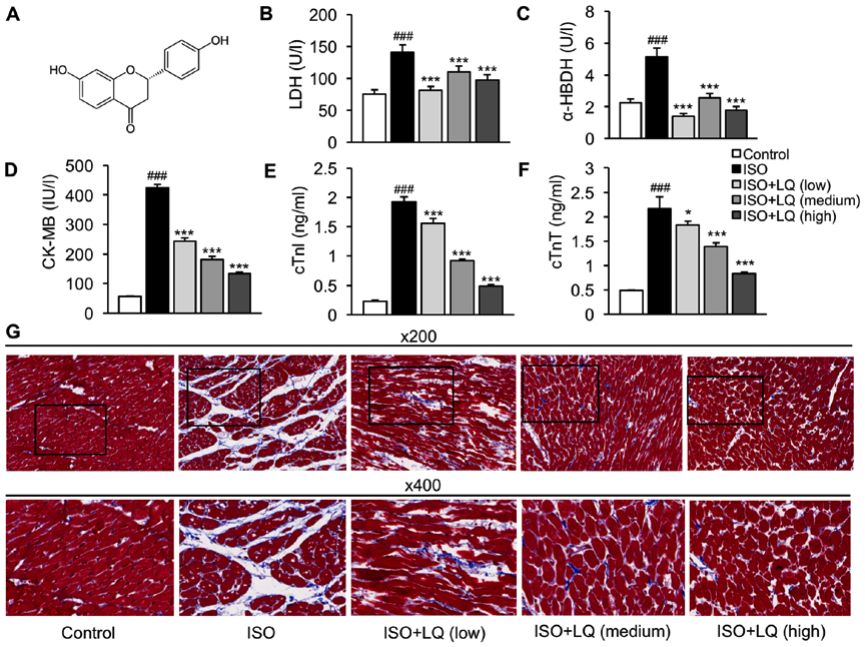
LQ ameliorates ISO-induced myocardial fibrosis in mice. (A) Chemical structure of LQ. (B-E) A mouse model of myocardial fibrosis was established. The serum levels of (B) LDH (U/l), (C) α-HBDH (U/l), (D) CK-MB (IU/l), (E) cTnl (ng/ml) and (F) cTnT (ng/ml) were measured by enzyme-linked immunosorbent assay. ^###^P<0.001 vs. the control group; *P<0.05, ***P<0.001 vs. the ISO group. (G) Mouse heart tissues were harvested for histopathological examination using Masson's trichrome staining. Representative images at ×200 and 400 magnification are shown. α-HBDH, α-hydroxybutyrate dehydrogenase; CK-MB, creatine kinase isoenzyme MB; cTnI, cardiac troponin I; cTnT, cardiac troponin T; ISO, isoprenaline; LDH, lactate dehydrogenase; LQ, liquiritigenin.

Liquiritigenin (LQ) is a flavanone compound with a polyphenolic structure (Fig. 1A), which is primarily found in the roots of licorice (*Glycyrrhiza glabra, Glycyrrhizae radix*). Previous evidence has demonstrated that LQ possesses multiple pharmacological and biochemical properties, including anti-oxidative, anti-carcinogenic, anti-inflammatory, hepatoprotective and estrogenic activities (10). Recently, the anti-fibrogenic role of LQ has been identified in a murine model of carbon tetrachloride-induced hepatic fibrosis via regulating the TGF-β1/Smad pathway (11). It has also been shown that LQ may attenuate high-glucose-induced ECM accumulation, inflammatory response and oxidative stress in rat glomerular mesangial cells (12). Moreover, LQ has been shown to exhibit cardioprotective effects against high fructose-induced cardiac injury in mice and cardiac muscle cells by suppressing the markers of fibrosis and inflammation, suggesting the therapeutic potential of LQ in diabetic heart injury (13). However, whether LQ could attenuate myocardial fibrosis and preserve cardiac function following heart injury remains unclear.

In the present study, the potential role of LQ in myocardial fibrosis was investigated in a mouse model induced by isoprenaline (ISO) and in an *in vitro* model stimulated with angiotensin II (Ang II). The effects of LQ on collagen deposition, cardiomyocyte damage and cardiac function were examined. In addition, the regulation of LQ in the TGF-β1/Smad2 and the AKT/ERK signaling pathways was investigated.

## Materials and methods

### 

#### Animals and study design

Male C57BL/6 mice (age, 6–8 weeks; weight, 23.7±1.2 g; strain code, 027) were purchased from Charles River Laboratories, Inc. The mice were housed in a pathogen-free facility under a 12-h light/dark cycle, at a temperature of 23±2°C and at 50% humidity. Animals had *ad libitum* access to food and water and were allowed to acclimate for at least 1 week prior to the study. The present study was approved by the Laboratory Animal Management and Welfare Ethical Review Committee of Zhejiang Traditional Chinese Medicine University (Hangzhou, China; approval no. ZSLL-2019-023) and all experiments were performed following the Guide for the Care and Use of Laboratory Animals (14).

Animals were randomly divided into the following five groups (n=6/group): Control, ISO, ISO + LQ (low), ISO + LQ (medium) and ISO + LQ (high). The model of ISO-induced myocardial fibrosis was established in all mice, with the exception of mice in the control group, as previously described (15). Mice subjected to ISO stimulation were subcutaneously injected with ISO (Sigma-Aldrich; Merck KGaA) at a dosage of 5 mg/kg on the first day, followed by 2.5 mg/kg/day for 2 weeks. Mice in the control group were treated with an equal volume of normal saline (Sigma-Aldrich; Merck KGaA) via subcutaneous injection following the same procedure. LQ (purity >98.9%) was purchased from Selleck Chemicals. Mice in the ISO + LQ (low), ISO + LQ (medium) and ISO + LQ (high) groups were intragastrically administered with LQ at a dosage of 10, 20 and 30 mg/kg/day, respectively, for 2 weeks prior to ISO stimulation and were continuously treated with LQ (10, 20 and 30 mg/kg/day, respectively) via intragastric administration during the period of ISO treatment.

#### Echocardiographic measurement

A total of 24 h post-treatment, echocardiography was performed in all groups of mice using a Vevo 770^®^ high-resolution imaging system (VisualSonics, Inc.) as previously described (16). Briefly, the mouse was positioned on a heating pad in a supine position. Anesthesia was induced with 5% isoflurane (Sigma-Aldrich; Merck KGaA) and maintained with 1.5% isoflurane. The chest of the mouse was shaved and acoustic coupling gel (Olympus Corporation) was applied to the surface of the thorax. When the heart rate was within the normal range, an M-mode cursor was positioned perpendicularly to the posterior walls of the left ventricle (LV). The echocardiograms of the LV in M mode, and the parameters including LV end-diastolic dimension (LV EDD, mm), LV end-systolic dimension (LV ESD, mm), fractional shortening (FS, %) and ejection fraction (EF, %), were obtained using M-mode ultrasound imaging. FS was calculated as follows: FS (%)=(LV EDD-LV ESD)/LV EDD ×100.

#### Assessment of myocardial injury markers

Following the measurement of echocardiographic parameters, all mice were anesthetized via intraperitoneal injection of pentobarbital sodium (35 mg/kg body weight; Sigma-Aldrich; Merck KGaA). Blood samples (1 ml) were collected from the abdominal vena cava, transferred to 10-ml centrifuge tubes and maintained at 4°C overnight. Subsequently, the mice were sacrificed via exsanguination under anesthesia. The serum samples were prepared by 30-min centrifugation of blood samples at 2,000 × g at 4°C. The serum levels of cardiac injury biomarkers were measured using commercial ELISA kits, including lactate dehydrogenase (LDH; cat. no. MBS2018912; MyBioSource, Inc.), α-hydroxybutyrate dehydrogenase (α-HBDH; cat. no. MBS9303787; MyBioSource, Inc.), creatine kinase isoenzyme MB (CK-MB; cat. no. A77886; Antibodies.com), cardiac troponin I (cTnI; cat. no. B52699; Beckman Coulter, Inc.) and cardiac troponin T (cTnT; cat. no. MBS 726068; MyBioSource, Inc.).

#### Histopathological analysis

Mouse heart tissues were harvested immediately after blood collection and transferred to Petri-dishes filled with cold normal saline. The adherent connective tissues and atrial appendage were carefully removed, and the apex was resected. All procedures were performed on ice. A portion of the apex was used for histopathological examination and the remaining portion was stored at −80°C for western blot analysis. For histopathology, heart tissues were fixed in 4% paraformaldehyde at 4°C for 24 h, embedded in paraffin and cut into 4-µm sections. Subsequently, Masson's trichrome staining (cat. no. ab150686; Abcam) was performed to examine morphological changes and to evaluate collagen deposition in cardiac tissues, following the manufacturer's instruction. Slides were observed under a polarized light microscope.

#### Cell culture

The rat myocardial cell line H9C2 (Merck KgaA; http://www.sigmaaldrich.com/FR/fr/product/sigma/cb_88092904) was cultured in DMEM containing 10% fetal bovine serum (Gibco; Thermo Fisher Scientific, Inc.) and F12 factor (Gibco; Thermo Fisher Scientific, Inc.) in an incubator containing 5% CO_2_ at 37°C. Ang II (Sigma-Aldrich; Merck KGaA) was used to establish an *in vitro* model of cardiac dysfunction in H9C2 cells. When cells had reached 70–80% confluence, they were digested with 0.25% trypsin (Gibco; Thermo Fisher Scientific, Inc.) and plated in 12-well plates at a density of 1×10^5^ per well. Following adhesion, cells in the Ang II + LQ (low), Ang II + LQ (medium) and Ang II + LQ (high) groups were pretreated with LQ at 0.1, 1 and 10 µM, respectively, for 6 h at 37°C followed by stimulation with Ang II (1 µM) for 24 h at 37°C. The Ang II group was pretreated with an equal volume of phosphate-buffered saline and then exposed to Ang II (1 µM) for 24 h. The control group remained untreated.

#### Flow cytometry

Apoptotic cell death was examined by flow cytometry as previously described (17). Briefly, H9C2 cells were digested, washed with cold PBS, centrifuged at 140 × g for 5 min at room temperature, and resuspended in 500 µl 1X binding buffer mixed with 5 µl Annexin V-FITC and 5 µl propidium iodide (apoptosis kit from Abcam) for 10 min at room temperature in the dark. The apoptosis of H9C2 cells was detected using a Navios flow cytometer and Kaluza software version 1.3 (both from Beckman Coulter, Inc.). The apoptosis rate was calculated as the number of early and late apoptotic cells over the number of total cells observed.

#### Western blot analysis

Heart tissue homogenates and cell lysates were prepared in RIPA buffer containing protease and phosphatase inhibitors (Bio-Rad Laboratories, Inc.). The protein content was measured using bicinchoninic acid assay (Pierce; Thermo Fisher Scientific, Inc.). Equal amounts of total protein (50 µg) from each sample were separated by 10% SDS-PAGE and then transferred to PVDF membranes (EMD Millipore). After blocking with 5% non-fat milk for 1 h at room temperature, membranes were incubated with the following primary antibodies at 4°C overnight: α-SMA (1:1,000; cat. no. ab5694; Abcam), Smad2 (1:800 dilution; cat. no. ab63576; Abcam), TGF-β1 (1:1,000; cat. no. ab92486; Abcam), collagen III (1:2,000 dilution; cat. no. ab7778; Abcam), collagen I (1:1,000 dilution; cat. no. ab34710; Abcam), AKT (1:800 dilution; cat. no. ab8805; Abcam), phosphorylated (p)-AKT (1:1,000 dilution; cat. no. 9271; Cell Signaling Technology, Inc.), ERK (1:1,000 dilution; cat. no. 4695; Cell Signaling Technology, Inc.), p-ERK (1:1,000 dilution; cat. no. 9101; Cell Signaling Technology, Inc.) and GAPDH (1:3,000 dilution; cat. no. ab181602; Abcam). After washing with TBS-0.1% Tween-20 buffer, the membranes were incubated with a secondary antibody (1:2,000 dilution; cat. no. ab6721; Abcam) at room temperature for 1 h. Blots were visualized using the BeyoECL Plus kit (Beyotime Institute of Biotechnology). The protein bands were semi-quantified using the Alphalmager 2000 Imaging System (ProteinSimple).

#### Statistical analysis

All data were analyzed using SPSS software (version 24.0; IBM Corp.) and are presented as the mean ± standard deviation from three independent experiments. One-way analysis of variance followed by Tukey's post-hoc analysis was used to determine statistical significance among the groups. P<0.05 was considered to indicate a statistically significant difference.

## Results

### 

#### LQ attenuates ISO-induced cardiomyocyte damage and myocardial fibrosis in mice

To investigate the potential regulatory effects of LQ on myocardial fibrosis, a murine model of ISO-induced myocardial fibrosis was established in C57BL/6 mice. Mice subjected to LQ treatment were intragastrically administered LQ at low (10 mg/kg/day), medium (20 mg/kg/day) and high (30 mg/kg/day) dosages, before and during the period of ISO stimulation. By measuring the levels of cardiac injury biomarkers in the serum samples, it was revealed that the ISO model group exhibited significantly increased levels of LDH, α-HBDH, CK-MB, cTnI and cTnT compared with those in the control group, whereas LQ treatment significantly suppressed the levels of these proteins at all doses tested (Fig. 1B-F). Furthermore, Masson's trichrome staining was performed to assess the morphological and pathological features of heart tissue samples from each group. The cardiomyocytes in the control group were compactly arranged without intercellular space, whereas in ISO-treated mice, cardiomyocyte arrangement was disordered and myocardial fibers were ruptured, which was accompanied by myocardial atrophy and inflammatory cell infiltration (Fig. 1G). Treatment with LQ ameliorated fibrosis and inflammation in ISO-induced hearts; the most robust effect was observed at a dosage of 30 mg/kg/day (Fig. 1G). These results suggested that LQ treatment attenuated ISO-induced cardiomyocyte damage and myocardial fibrosis.

#### LQ improves cardiac function in ISO-treated mice

**Figure 2.**
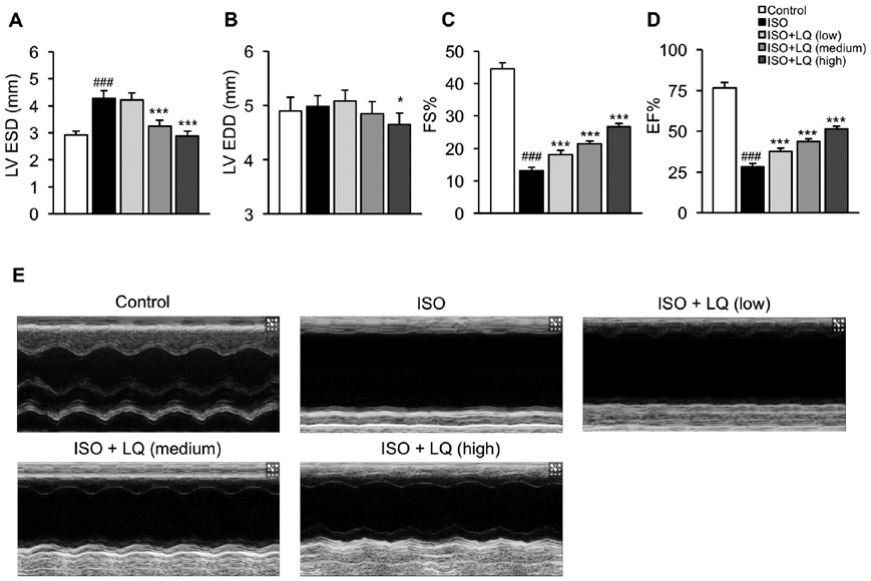
Effect of LQ on cardiac function in ISO-treated mice. Echocardiography was performed in all groups of mice using an M-mode ultrasound imaging system. The parameters indicating cardiac function were measured, including (A) LV ESD (mm), (B) LV EDD (mm), (C) FS (%) and (D) EF (%). ^###^P<0.001 vs. the control group; *P<0.05, ***P<0.001 vs. the ISO group. (E) M-mode echocardiograms of the LV from each group of mice are shown. EF, ejection fraction; FS, fractional shortening; ISO, isoprenaline; LQ, liquiritigenin; LV, left ventricle; LV EDD, LV end-diastolic dimension; LV ESD, LV end-systolic dimension.

To determine whether LQ could improve the cardiac function of mice treated with ISO, the LV internal dimensions (LV ESD and LV EDD) and ejection phase indices (FS and EF) of all groups of mice were measured by echocardiography. Compared with that in the control group, mice treated with ISO exhibited a significant increase in LV ESD, but not LV EDD, whereas LQ at medium and high concentrations significantly decreased LV ESD in ISO-treated mice (Fig. 2A and B). In addition, mice treated with a high dosage of LQ showed significantly smaller LV EDD compared with that in the ISO model group (Fig. 2B). The FS and EF values in ISO-treated mice were significantly lower compared with those in the control group, whereas treatment with LQ effectively increased FS and EF in a dose-dependent manner (Fig. 2C and D). The M-mode echocardiograms of the LV also showed that LQ improved myocardial contractile function in ISO-induced mice (Fig. 2E). These findings indicated that LQ treatment ameliorated ISO-induced cardiac dysfunction in mice.

#### LQ suppresses activation of the TGF-β1/Smad2 and AKT/ERK pathways in mice treated with ISO

**Figure 3.**
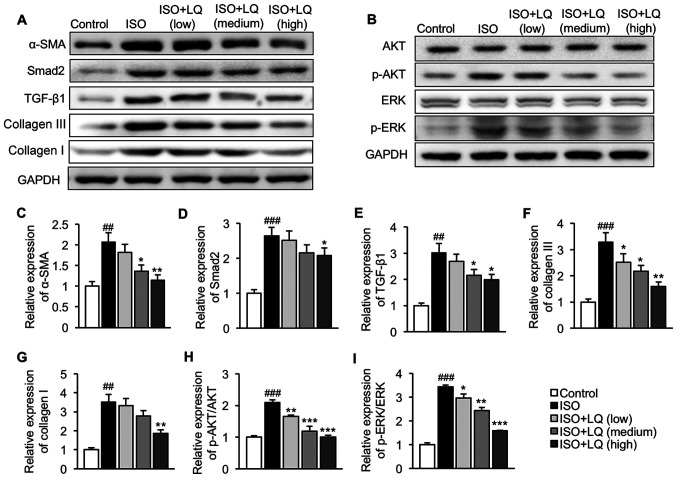
Effect of LQ on activation of the TGF-β1/Smad2 and AKT/ERK pathways in ISO-treated mice. Expression levels of proteins related to the (A) TGF-β1/Smad2 and (B) AKT/ERK pathways were measured by western blotting. Semi-quantification of the expression levels of (C) α-SMA, (D) Smad2, (E) TGF-β1, (F) collagen III, (G) collagen I, (H) p-AKT/AKT and (I) p-ERK/ERK normalized to the expression of GAPDH. ^##^P<0.01, ^###^P<0.001 vs. the control group; *P<0.05, **P<0.01, ***P<0.001 vs. the ISO group. AKT, protein kinase B; α-SMA, α-smooth muscle actin; ERK, extracellular signal-regulated kinase; ISO, isoprenaline; LQ, liquiritigenin; p-, phosphorylated; TGF- β1, transforming growth factor β1.

The present study further investigated the effects of LQ on regulation of the TGF-β1/Smad2 and AKT/ERK pathways. Mice in the ISO model group exhibited significantly upregulated α-SMA, Smad2, TGF-β1 collagen III and collagen I expression levels compared with those in the control group, indicating that ISO induced activation of the TGF-β1/Smad2 pathway in the heart (Fig. 3A and C-G). The intragastric administration of LQ at 30 mg/kg/day significantly decreased the expression levels of all these proteins (Fig. 3A and C-G). No significant difference was observed in the basal levels of AKT and ERK among all groups, whereas ISO significantly enhanced the expression levels of p-AKT and p-ERK as compared with those in the control mice, suggesting that activation of the AKT/ERK signaling pathway was induced in mice subjected to ISO stimulation (Fig. 3B, H and I). Treatment with LQ at 10, 20 and 30 mg/kg/day significantly inhibited the phosphorylation of both AKT (Fig. 3B and H) and ERK (Fig. 3B and I) in ISO-treated mice. These data supported the conclusion that LQ suppressed ISO-induced activation of the TGF-β1/Smad2 and AKT/ERK pathways.

#### LQ decreases the Ang II-induced apoptosis of cardiomyocytes

**Figure 4.**
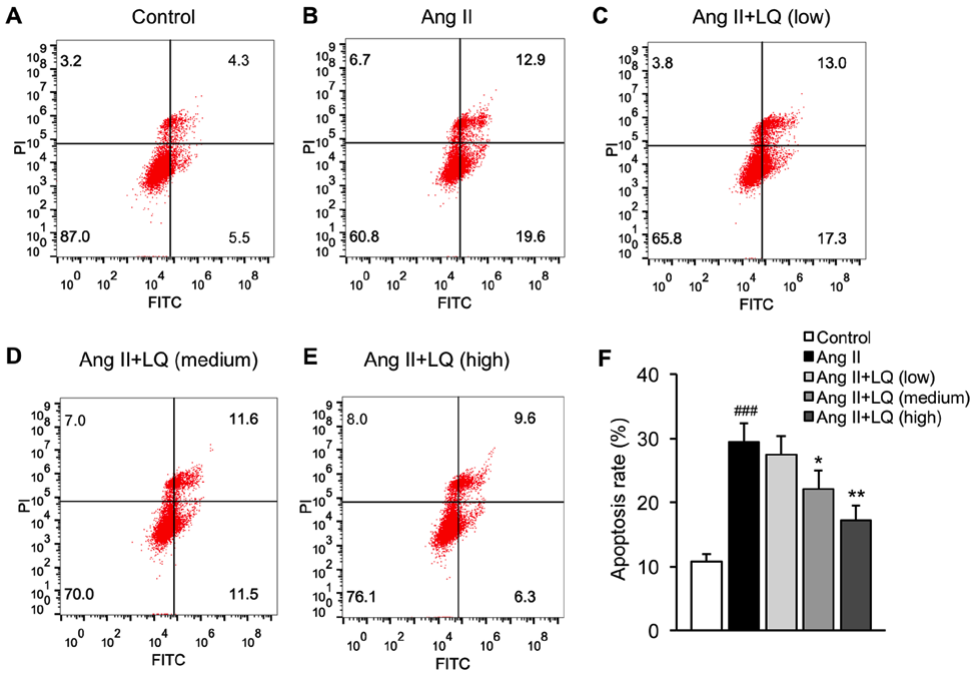
Effect of LQ on the apoptosis of cardiomyocytes following Ang II exposure. An *in vitro* model of cardiac dysfunction was established in rat myocardial H9C2 cells. Apoptosis of H9C2 cells in the (A) control, (B) Ang II, (C) Ang II + LQ (low), (D) Ang II + LQ (medium) and (E) Ang II + LQ (high) groups was examined by flow cytometry. (F) Apoptosis rate was calculated. ^###^P<0.001 vs. the control group; *P<0.05, **P<0.01 vs. the Ang II group. Ang II, angiotensin II; LQ, liquiritigenin; PI, propidium iodide.

The present study established a model of Ang II-induced cardiac dysfunction in rat myocardial H9C2 cells to examine the effects of LQ on cardiomyocyte apoptosis. Cells exposed to Ang II exhibited significantly increased apoptosis compared with that in the untreated group (Fig. 4A, B and F). Pretreatment with LQ at medium and high doses successfully decreased the apoptosis rate of H9C2 cells following Ang II treatment (Fig. 4C-F). These results indicated the anti-apoptotic effects of LQ on cardiomyocyte apoptosis.

#### LQ inhibits activation of the TGF-β1/Smad2 and AKT/ERK pathways in Ang II-induced cardiomyocytes

**Figure 5.**
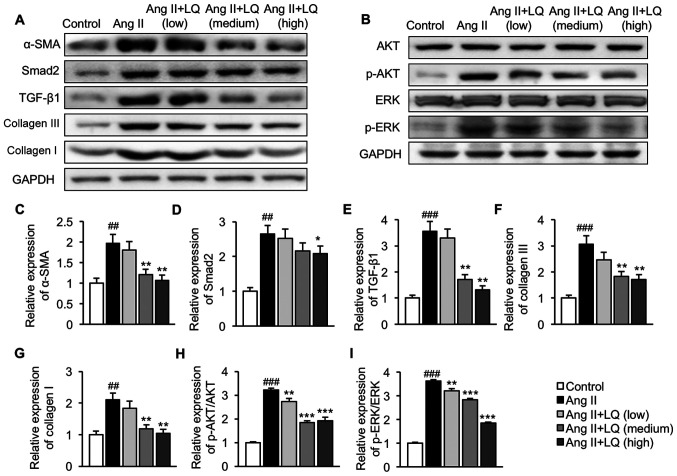
Effect of LQ on Ang II-induced activation of the TGF-β1/Smad2 and AKT/ERK pathways. H9C2 cells were collected following LQ pretreatment and Ang II stimulation. Cell lysates were prepared for western blot analysis. The expression levels of proteins related to the (A) TGF-β1/Smad2 and (B) AKT/ERK pathways were assessed. Semi-quantification of the protein expression levels of (C) α-SMA, (D) Smad2, (E) TGF-β1, (F) collagen III, (G) collagen I, (H) p-AKT/AKT and (I) p-ERK/ERK normalized to the expression of GAPDH. ^##^P<0.01, ^###^P<0.001 vs. the control group; *P<0.05, **P<0.01, ***P<0.001 vs. the Ang II group. AKT, protein kinase B; Ang II, angiotensin II; α-SMA, α-smooth muscle actin; ERK, extracellular signal-regulated kinase; LQ, liquiritigenin; p-, phosphorylated; TGF- β1, transforming growth factor β1.

Finally, the effect of LQ treatment on regulation of the TGF-β1/Smad2 and AKT/ERK pathways were evaluated *in vitro*. Consistent with the results obtained from the mouse ISO model, cells treated with Ang II exhibited significantly elevated expression levels of α-SMA, Smad2, TGF-β1, collagen III and collagen I compared with those in untreated cells, suggesting activation of the TGF-β1/Smad2 pathway in Ang II-induced cardiomyocytes (Fig. 5A and C-G). Pretreatment with LQ at 1 and 10 µM significantly decreased the protein expression levels of α-SMA, TGF-β1, collagen III and collagen I in H9C2 cells following Ang II exposure, whereas the upregulation of Smad2 in Ang II-treated cells was only significantly suppressed by LQ at the highest dosage (Fig. 5A and C-G). In addition, Ang II induced the phosphorylation of AKT and ERK in H9C2 cells without affecting the basal levels of AKT and ERK (Fig. 5B, H and I). A 6-h pre-incubation with 0.1, 1 and 10 µM LQ effectively reduced the phosphorylation of AKT (Fig. 5B and H) and ERK (Fig. 5B and I) in Ang II-stimulated H9C2 cells. Taken together, these results suggested that LQ served an inhibitory role in Ang II-induced activation of the TGF-β1/Smad2 and AKT/ERK pathways in cardiomyocytes.

## Discussion

Myocardial fibrosis is a fundamental process in ventricular remodeling and is considered a primary contributor to the progression of heart failure (18). Novel treatment strategies that collectively target the key signaling pathways and molecular factors involved in fibrosis need to be considered in the development of anti-fibrotic therapies (19). The present study demonstrated that LQ attenuated myocardial fibrosis in mice and cultured myocardial cells via regulating the TGF-β1/Smad2 and AKT/ERK signaling pathways, suggesting the protective effects of LQ on cardiac fibrosis.

The subcutaneous injection of ISO, a β-adrenergic agonist, has been reported to induce cardiac dysfunction and fibrosis in mice, which enables the evaluation of anti-fibrotic agents *in vivo* (20). CK-MB and cardiac troponins, as well as LDH and α-HBDH, are classic diagnostic markers that are highly sensitive and specific for the detection of myocardial damage in various conditions, including myocardial fibrosis (21). In the present study, the serum levels of LDH, α-HBDH, CK-MB, cTnI and cTnT in the ISO model group were significantly higher compared with those in the control group, whereas LQ treatment before and during ISO stimulation downregulated the levels of cardiac injury-related proteins. Further histological examination of mouse heart tissues revealed that LQ ameliorated ISO-induced ECM accumulation, myocardial disarray and inflammatory cell infiltration, further confirming the cardioprotective role of LQ in cardiac fibrosis. Previous evidence has suggested an association exists between the disruption of ECM homeostasis and impaired LV function (22). Using echocardiography, the present study compared the indicators of LV function in all groups of mice. The results revealed that the LV ESD and ejection phase indices in ISO-treated mice were significantly different from those in the control group, whereas LQ treatment effectively diminished the effects of ISO on these cardiac function markers.

The fibrotic response in the heart is a dynamic process in which pro-fibrotic factors, such as TGF-β1, are upregulated and trigger the activation of downstream signaling, including Smad, AKT and ERK (23). These pathways further induce the expression of fibrogenic genes (i.e. α-SMA and collagen), leading to excessive production of matrix metalloproteinases and subsequent ECM deposition (24). The present study revealed that ISO treatment increased cardiac expression of α-SMA, Smad2, TGF-β1, collagen III and collagen I compared with that in the control group. The treatment of ISO-treated mice with LQ at 30 mg/kg/day significantly decreased collagen formation and suppressed the TGF-β1/Smad2 pathway. Moreover, LQ at all concentrations inhibited ISO-induced phosphorylation of AKT and ERK with no effects on the basal levels of these proteins.

The apoptosis of cardiomyocytes is a major pathological event in myocardial fibrosis and has been regarded as a key mechanism contributing to impaired LV performance (25). Ang II is a bioactive compound in the renin-angiotensin system, which has been reported to accelerate myocardial remodeling and cardiac fibrosis by activating reactive oxygen species (ROS)-dependent pathways in rat cardiomyocytes (26). A recent study reported that Ang II exposure decreased the viability of H9C2 cells and stimulated pro-apoptotic signaling pathways by enhancing ROS production (27). The present study investigated the effect of LQ on the Ang II-induced apoptosis of cardiomyocytes. Pretreatment with LQ at medium and high dosages significantly reduced apoptotic cell death in H9C2 cells following Ang II treatment. Furthermore, the treatment of H9C2 cells with LQ inhibited Ang II-induced activation of the TGF-β1/Smad2 and AKT/ERK pathways in cardiomyocytes, which was consistent with the findings in the animal model. However, the use of the rat myocardial cell line H9C2 instead of primary cardiac fibroblasts may be considered as a limitation to the present study. Further investigations using primary cardiac fibroblasts are required to validate the current findings.

Taken together, the present study demonstrated that LQ ameliorated ISO-induced myocardial fibrosis in a mouse model and inhibited the apoptosis of cardiomyocytes *in vitro* by inhibiting both the TGF-β1/Smad2 and AKT/ERK signaling pathways. These findings elucidated the mechanisms underlying the anti-fibrotic and cardioprotective potential of LQ in fibrosis, and provided evidence to support further investigations into LQ for the management of cardiomyocyte injury and myocardial fibrosis in patients suffering from heart diseases.

## Data Availability

The datasets used and/or analyzed during the current study are available from the corresponding author on reasonable request.
